# Foresight for policy – addressing the challenges for policy impact

**DOI:** 10.1093/eurpub/ckac129.203

**Published:** 2022-10-25

**Authors:** L Bontoux

**Affiliations:** JRC, ISPRA, Italy

## Abstract

**Background:**

The role of policymaking is to create the future that we want. In its communication on Better Regulation, the European Commission recognises the need for strategic foresight to play a key role in helping to ‘future-proof’ EU policymaking. The aim is to ensure that decisions are grounded in a longer-term perspective. In times of rapid change, EU policymaking needs to have impact assessments, fitness checks and major regulatory evaluations informed by foresight. Strategic foresight is also a powerful way to engage with stakeholders and not only capture their perspectives, but also generate collective intelligence in the areas of policy interest.

**Lessons from EU policy making:**

This fits within a broader effort at the Commission to be at the forefront of excellence in policymaking in Europe and worldwide. This is why the EU Policymaking Hub was launched 2020. It offers a platform for policymakers to learn, collaborate and share knowledge in EU policymaking, introducing new capacity building offers. It aims at strengthening the Commission's policymaking capacity through anticipating, developing, implementing, monitoring, and evaluating policies in an evidence-informed, transparent, and collaborative way with stakeholders, citizens and experts. Providing the policymaking community with a framework for long-term competence development will strengthen the profession, make it fit for the future, contribute to colleagues’ motivation and help the Commission to achieve its goals.

**Conclusions:**

Being futures literate covers a range of skills from anticipation to the ability to run in-depth foresight processes. It spans a range of abilities from having an anticipatory mindset to scanning for change, understanding change and being able to influence change. Since its inception, this competence framework is at the base of an effort to train European civil servants in foresight and to increase foresight literacy for application in policymaking.

